# Oxford nanopore sequencing as a useful tool for investigating the population dynamics of invasive begomoviruses in Sicily

**DOI:** 10.1099/mgen.0.001529

**Published:** 2025-11-03

**Authors:** Sofia Bertacca, Silvia Rotunno, Fulco Frascati, Emanuela Noris, Gian Paolo Accotto, Salvatore Davino, Laura Miozzi, Anna Maria Vaira

**Affiliations:** 1 Institute for Sustainable Plant Protection, National Research Council, Torino 10135 , Italy; 2 Department of Agricultural, Food and Forest Sciences (SAAF), University of Palermo, Palermo 90128 , Italy

**Keywords:** geminivirus, MinION platform, tomato, TYLCV-IMS54 recombinant, TYLCV recombinants

## Abstract

Tomato yellow leaf curl disease is a major viral disease severely affecting tomato crops in the Mediterranean region, leading to reduced crop yield and significant economic losses. The disease is caused by monopartite begomoviruses belonging to the *Geminiviridae* family, primarily tomato yellow leaf curl Sardinia virus (TYLCSV) and tomato yellow leaf curl virus (TYLCV), which often co-infect tomato plants, promoting the emergence of recombinant viral genomes. To investigate the diversity and evolutionary dynamics of these viruses, symptomatic plants collected from agricultural sites in Sicily between 2020 and 2022, along with archived plant samples from 1994 to 1999, were analysed. For each collection site, leaves from symptomatic plants were pooled to form representative samples. Total nucleic acids were extracted and subjected to rolling circle amplification to enrich circular viral genomes. The amplified products were sequenced using Oxford Nanopore Technologies (ONT) long-read sequencing to obtain full-length viral genomes. Bioinformatic analyses revealed that archived samples mainly contained TYLCSV-related sequences, confirming its historical predominance in Sicilian agroecosystems. Recent samples, by contrast, were dominated by TYLCV-derived recombinants such as TYLCV-IS141- and TYLCV-IS76-like variants, indicating a temporal shift in the structure of the viral population. Furthermore, a distinct group of newly emerged recombinants, provisionally referred to as TYLCV-IMS54, was identified in the most recent samples. Their genome comprises a TYLCV backbone, a 54-nt segment from TYLCSV located downstream of the stem-loop region and a 341-nt region derived from TYLCV-Mild. These results demonstrate the importance of continuous viral population monitoring through ONT-based sequencing to detect emerging variants that may influence disease management strategies in tomato crops and highlight the central role of recombination in shaping begomovirus populations.

**Impact Statement**

Tomato yellow leaf curl disease (TYLCD) is one of the most damaging viral diseases affecting tomato crops in the Mediterranean basin, yet the long-term dynamics of its causal agents and the role of recombination remain challenging due to the genome plasticity of these viruses. This study provides an updated and comprehensive picture of the begomovirus population structure in Sicily, a key agricultural region for tomato production, by analysing both contemporary and historical plant samples. Through the application of Oxford Nanopore Technologies (ONT) long-read sequencing combined with rolling circle amplification and bioinformatic analyses, this research identified persistent recombinant genotypes including a distinct group of newly emerged recombinants, named TYLCV-IMS54. These findings expand current knowledge on the genetic variability and evolutionary processes shaping begomovirus populations in Sicilian agroecosystems. The detection of recombinant genomes highlights the enduring role of recombination in begomovirus diversification. By integrating sequencing data with population and phylogenetic analysis, this work offers valuable insights into the epidemiology and management of TYLCD in regions heavily impacted or newly colonized by these viral pathogens. The study also underscores the importance of continuous molecular surveillance using ONT-based platforms to enable early detection of emerging recombinant variants, with significant implications for plant virology, crop protection and agricultural biosecurity strategies.

## Data Summary

Raw reads are deposited in the Sequence Read Archive of National Center for Biotechnology Information (NCBI) (https://www.ncbi.nlm.nih.gov/sra) with BioProjects ID PRJNA1226414 and PRJNA1273745; the recombinant TYLCV-IMS54 sequence is available in GenBank with accession number PQ873011. Parental TYLCV strains used in this study could be retrieved in GenBank with the following accession nos: DQ144621 for TYLCV, NC_003828 for TYLCSV and KJ913682 for TYLCV-Mild. Recombinant strains could be retrieved in GenBank with the following accession nos.: LN846609 for TYLCV-IS76 and AF271234 for TYLCMaV. The authors confirm that all supporting data, code and protocols have been provided within the article or through supplementary data files.

## Introduction

Tomato yellow leaf curl disease (TYLCD) is a major disease of tomato crops, causing significant economic losses worldwide. TYLCD is induced by viruses of the genus *Begomovirus* (family *Geminiviridae*) [1], one of the largest and most economically important groups of plant-infecting viruses, transmitted in a persistent-circulative manner by the whitefly *Bemisia tabaci*. The rapid spread of TYLCD is exacerbated by the dynamic nature of the geminivirus genomes, which exhibit high mutation and recombination rates that enable them to rapidly adapt to new hosts and environmental conditions [2]. In Italy, two predominant TYLCD-associated species have been identified: tomato yellow leaf curl Sardinia virus (TYLCSV) (*Begomovirus solanumflavusardiniaense*), first reported in Sardinia in 1989 [3,4], and tomato yellow leaf curl virus (TYLCV) (*Begomovirus coheni*), detected in Sicily in 2002 [5]. The co-existence of these two species resulted in the emergence of various recombinants, highlighting a high evolutionary flexibility [6]. The widespread cultivation of tomato varieties carrying resistance genes, particularly *Ty-1*, further shaped viral population dynamics. Indeed, while these resistance genes initially provided effective control of the disease, they exerted a selective pressure on the viral population, leading to the appearance of resistance-breaking variants [7]. Understanding the genetic diversity and adaptability of these viruses requires robust tools capable of capturing the complexity of their genomic evolution. To identify TYLCD-related isolates and recombinants, various diagnostic techniques are employed, each with specific advantages in sensitivity, specificity and applicability, ranging from restriction fragment length polymorphism analysis followed by polyacrylamide gel electrophoresis and hybridization-based approaches [8,9], to more advanced molecular techniques such as singleplex or multiplex PCR [10,11], loop-mediated isothermal amplification (LAMP) [12], and the combination of LAMP with CRISPR-Cas [13]. Recent breakthroughs in sequencing technologies are continuously revolutionizing the study of viral diversity, evolution and recombinant detection [14]. In particular, the Oxford Nanopore Technologies (ONT) MinION platform has emerged as a powerful tool for the real-time identification of viral genomes [14]. Its capacity to generate long-read sequences facilitates precise detection of genetic variations, including mutations and recombination events, which are key drivers of viral evolution [15]. Furthermore, the portability and cost-effectiveness of ONT MinION make it a valuable tool for field-based applications and rapid diagnostics, enabling researchers to address challenges in TYLCD management with unprecedented efficiency [16].

In this study, using the combination of rolling circle amplification (RCA) and the ONT MinION platform, we provide a comprehensive overview of the genetic diversity and population dynamics of TYLCD-associated viruses in tomato and cucurbit plants across Sicily, a Southern Italian region where this disease causes significant agricultural losses. The evolutionary mechanisms underlying the adaptation of these viral populations are discussed.

## Methods

### Sample collection and DNA extraction

**Fig. 1.**
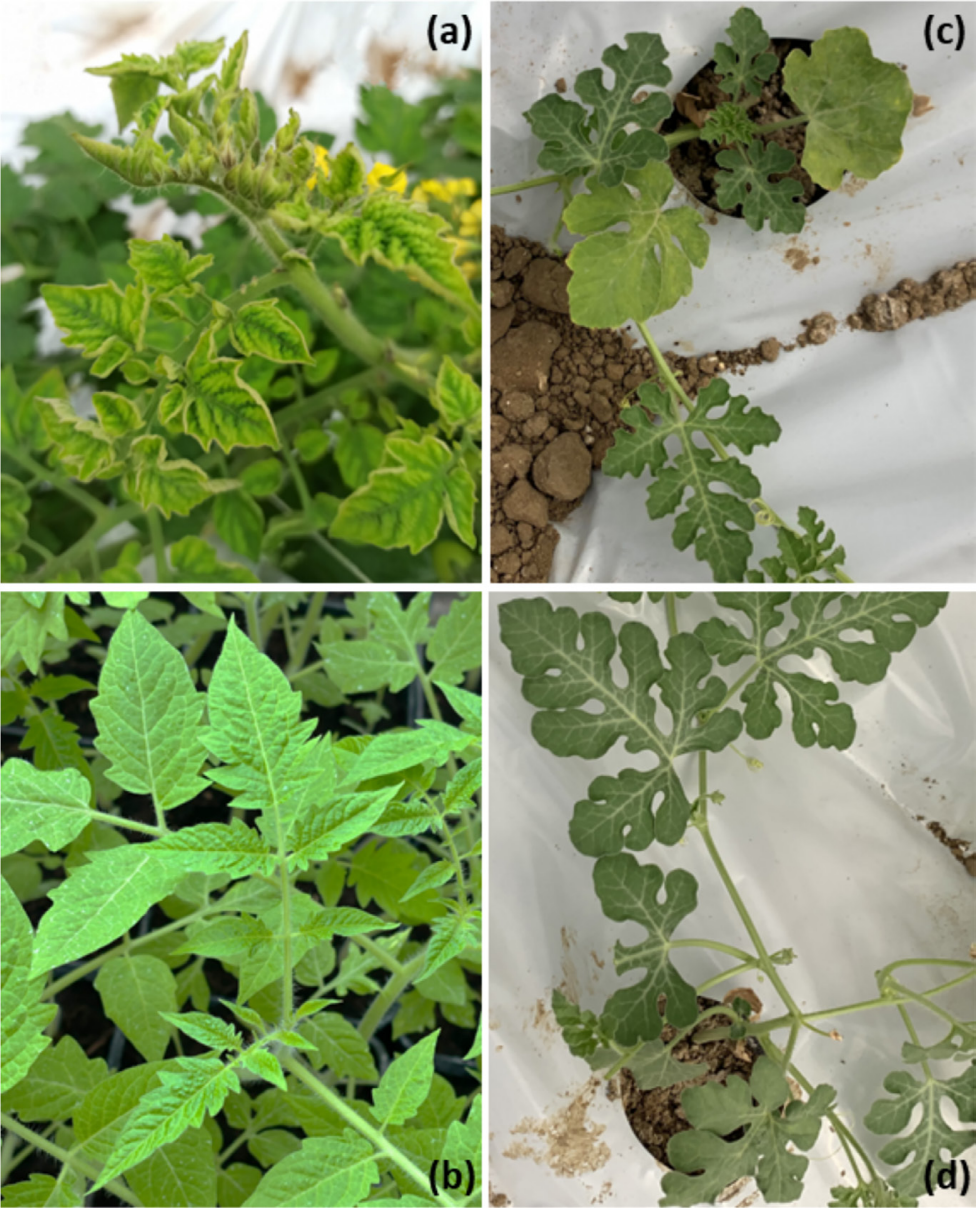
Geminiviral symptoms in plants tested in this work. (**a**) Tomato plant var. Demetrio (TYLCV tolerant) (sample 141122_t_1) showing typical yellowing, leaf cupping and deformation in the plant apex. (**b**) Healthy tomato plant. (**c**) Watermelon plant showing chlorosis, mosaic and stunting in the basal leaves (sample 150721_w_1). (**d**) Healthy watermelon plants.

During the years 2020–2022, five tomato (*Solanum lycopersicum*) fields showing TYLCD symptoms in the Ragusa province and one watermelon (*Citrullus lanatus*) field with atypical strong leaf mosaic symptoms in the Agrigento province were visited (Fig. 1). From each field, young leaves from 20 randomly selected symptomatic plants were collected and pooled to create a single sample representing the whole field. Total nucleic acids were extracted from each sample (Table 1) using the previously described dot-blot method [17]. Nucleic acid concentration was quantified using a NanoDrop spectrophotometer (Thermo Scientific, Wilmington, USA). Additionally, DNA extracted from tomato and weed samples collected in the Ragusa province between 1994 and 1999 and archived at −20 °C in our laboratory was used to investigate the evolutionary dynamics of TYLCD.

**Table 1.** List of samples analysed by MinION sequencing, consisting of pools of symptomatic leaves collected in Sicily in the years 1994–2022

Sampling year	Sample ID	MinION code	Location	Species/cultivar	Genotype	No. of plants pooled in the sample
1994	051294_vv	D1K8_016	RG	*Malva* spp., *Chenopodium album*, *Potentilla* spp., *Portulaca* spp. and other weeds	N.A.	10
1999	130999_t_1	D1K8_017	RG	Tomato	U	10
	130999_t_2	D1K8_018	RG	Tomato	U	10
2020	061020_t_2	HPCT001	Acate (RG)	Tomato/Creativo	T	20
	071020_t_3	HPCT002	Scicli (RG)	Tomato/Cikito	S	20
2021	141021_t_4	D1K8013	Vittoria (RG)	Tomato/Cherry	T	20
	141021_t_5	D1K8007	Vittoria (RG)	Tomato/Datterino	U	20
	150721_w_1	D1K8010	Licata (AG)	Watermelon	U	20
2022	141122_t_1	D1K8009	Vittoria (RG)	Tomato/Demetrio	T	20

AG, Agrigento; N.A., not applicable; RG, Ragusa; RG, Ragusa; S, susceptible; T, tolerant; T, tolerant; U, unknown.

### Rolling circle amplification

To enrich circular DNA molecules, RCA was performed on total nucleic acid extracts using the TempliPhi™ 100 Amplification Kit (Cytiva 25-6400-10, Sigma-Aldrich, St. Louis, MO, USA), running the reaction at 30 °C for 30 h. One-tenth volume of the RCA product was digested with either *BamH*I or *EcoR*I enzymes; linearized DNAs were resolved by electrophoresis on 1% agarose gels in 0.5X TBE buffer and visualized following staining with RedSafe™ (iNtRON Biotechnology, Korea), using undigested RCA products as a control. The latter was precipitated with three volumes of ice-cold ethanol, and 0.1 vol of 3M sodium acetate and DNA was resuspended in 20 ul of sterile water and quantified by NanoDrop spectrophotometer.

### MinION sequencing and recombination analysis

Undigested RCA products were submitted to a specialized sequencing company (MicrobesNG, Birmingham, UK) for long-read sequencing on the ONT MinION platform, following the manufacturer’s instructions. The MicrobesNG procedure included long-read library preparation using the Rapid Barcoding Kit 96 V14 (SQKRBK114.96) and sequencing with a R10.4.1 flowcell (FLO-MIN114) on GridION. Raw signal data were basecalled using the GridION deployment for Guppy (ont-guppy-for-gridion v. 6.3.9) using model number r1041_e82_400bps_hac_v4.2.0 with barcode trimming enabled. Reads under 200 bp were discarded. Raw long reads obtained from MicrobesNG were converted from FASTQ to FASTA format using Seqtk (v. 1.4-r130-dirty). Consensus sequences were assembled with TideHunter (v. 1.5.5) [18]. Sequences were subjected to a blastn search against a custom database comprising 16,957 *Geminiviridae* sequences downloaded from the NCBI Virus Database (https://www.ncbi.nlm.nih.gov/labs/virus/vssi/#/, accessed on 19 May 2025, filters applied: GenBank and complete). For recombination analysis, geminivirus-related sequences longer than 2,770 nt were checked with ORFfinder (https://www.ncbi.nlm.nih.gov/orffinder/) and multi-aligned with muscle in mega11 (v. 11.0.13) [19] with putative parental TYLCV-related genomic sequences. The sequence demarcation tool (SDT) (v. 1.3) [20] was used to classify virus sequences based on sequence pairwise identity. Recombination events were subsequently identified using the Recombination Detection Program (RDP) (v. 4.101) [21]. Recombination events have been accepted only if identified by at least five different methods. Representative sequences of each recombination event identified by RDP were verified using blastn and used for phylogenetic analysis.

### End-point PCR analysis

Recombination breakpoints were validated by end-point PCR. Regions useful to design specific primers were selected following alignment of viral genomic sequences using clustalw within BioEdit (v. 7.7.1) [22]. Primer specificity was assessed using nucleotide blast (https://www.ncbi.nlm.nih.gov, accessed on 7 June 2025), and the secondary structure was verified with the OligoAnalyzer Tool (https://eu.idtdna.com/calc/analyzer, accessed on 7 June 2025). The primer pair IMS54-2592-F (5′-GGAAVCGCTTAGGAGGAGCCAT-3′) and IMS54-138-R (5′-TTGCAAVACAAATTACTTGGGGA-3′) was designed to selectively amplify a region of 329 nt. The assay included as control the following isolates: DQ144621 for TYLCV, NC_003828 for TYLCSV, and KJ913682 for TYLCV-Mild, LN846609 for TYLCV-IS76, and AF271234 for TYLCMaV. DNAs from healthy tomato and cucurbit plants were used as negative controls. PCR was performed using Platinum™ II Taq Hot-Start DNA Polymerase Kit (Invitrogen), following the manufacturer’s instructions, with an annealing temperature of 58 °C. PCR products were separated by electrophoresis as above. Selected PCR products were purified using the DNA Clean and Concentrator kit (Zymo Research) for Sanger sequencing analysis (BMR Genomics S.r.l., Padova, Italy).

### Phylogenetic analyses

To construct the phylogenetic tree, parental TYLCV strains and recombinants from the Mediterranean area were downloaded from NCBI Virus (accessed on 23 May 2025) and multi-aligned with the newly identified representative recombinant sequences using muscle in mega11. A neighbour-joining (NJ) tree with 1,000 bootstrap replicates was reconstructed with mega11. The sequence similarity level was estimated with blastn.

## Results

### Identification of TYLCD-related genomic sequences

Using the ONT MinION platform combined with RCA, between 91 and 412 consensus sequences were obtained for each of the nine samples analysed, as detailed in Table 2. To identify TYLCD-related sequences, reads longer than 2,770 nt were selected and used as input for a blastn search against the *Geminiviridae* custom database. Several TYLCD-related sequences (2 to 135) were identified. Sequences showing identity with TYLCD genomes were checked with ORFfinder to identify coding regions and only those with correct ORFs (2 to 50) were retained for further analysis (Tables 2 and S1, available in the online Supplementary Material).

**Table 2.** ONT MinION long reads statistics

Sample ID	Sample	Raw read	Cleaned read	Consensus sequence	Consensus sequences >2,770 nt	TYLCD-related sequence	TYLCD-related sequences with correct ORF
HPCT_001	Tomato	4,130	2,094	119	35	35	2
HPCT_002	Tomato	3,144	1,597	91	22	22	2
D1K8_007	Tomato	2,280	1,152	202	80	78	10
D1K8_009	Tomato	4,011	2,029	219	33	28	2
D1K8_013	Tomato	2,371	1,348	218	39	26	5
D1K8_010	Watermelon	3,644	1,831	230	8	2	2
D1K8_016	Weeds	2,425	1,220	412	20	4	1
D1K8_017	Tomato	2,299	1,153	293	138	135	44
D1K8_018	Tomato	2,528	1,269	277	129	122	50

### Recombination analysis of samples collected in 2020–2022

**Fig. 2.**
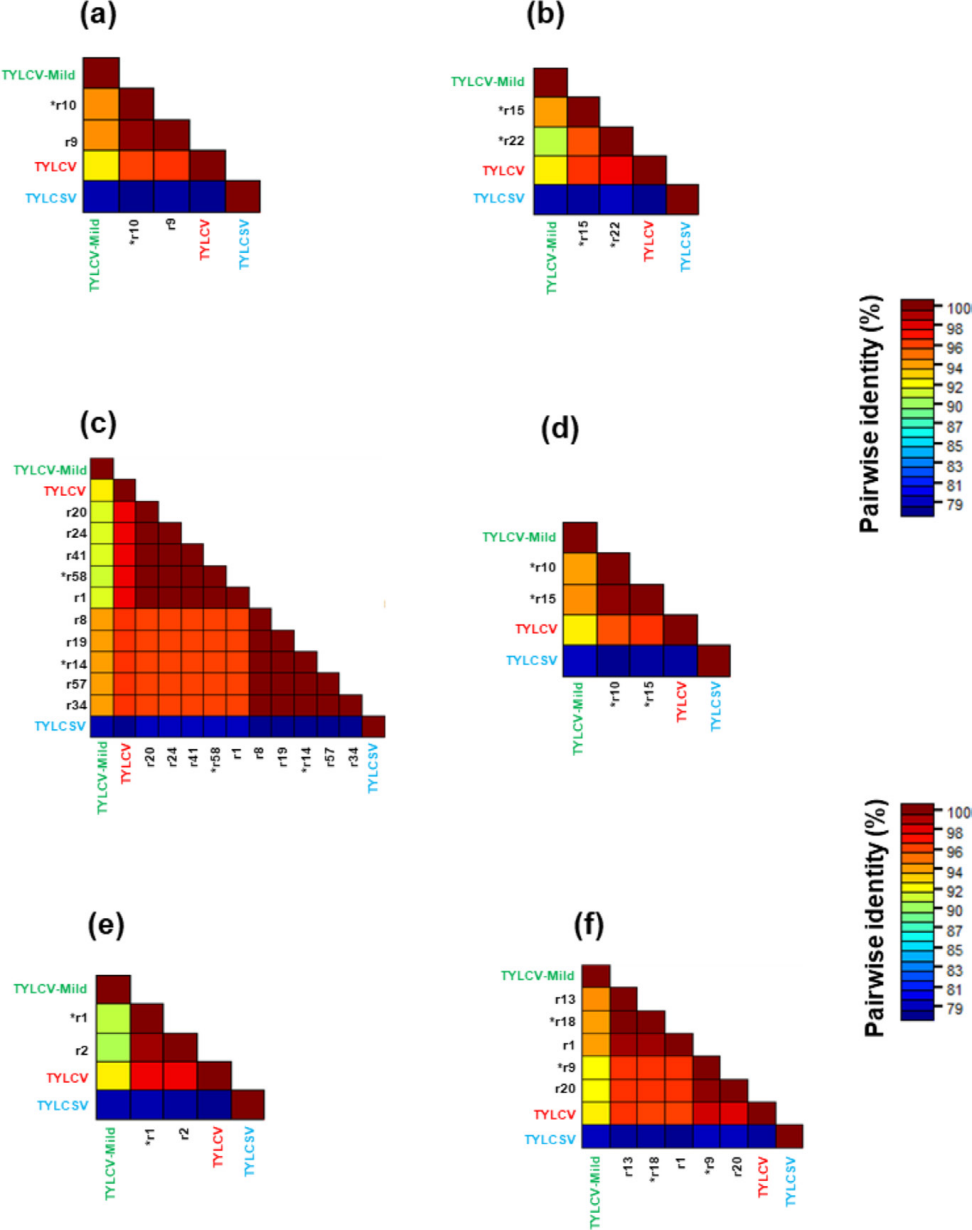
Pairwise identity matrices of TYLCD-related sequences obtained from each sample and the parental sequences from GenBank. Each matrix represents individual samples as follows: (**a**) HPCT_001, (**b**) HPCT_002, (**c**) D1K8_007, (**d**) D1K8_009, (**e**) D1K8_010 and (**f**) D1K8_013. Recombinant (**r**) sequences were named with progressive numbers, within each sample. Parental genomes are written according to the following colour code: TYLCV in red, TYLCV-Mild in green and TYLCSV in blue. Different clusters are identified at ≥94% identity, the defined value for strain demarcation of begomoviruses. Representative sequences for each cluster used for phylogenetic analysis are indicated with an asterisk.

Following alignment with the parental genomes, TYLCD-related sequences obtained from samples collected from 2020 to 2022 (hereafter referred to as r followed by a progressive number) showed levels of similarity to TYLCV or TYLCV-Mild higher than 94%, a value proposed as a strain demarcation value for the *Begomovirus* genus by the *Geminiviridae* Study Group of the International Committee on Taxonomy of Viruses [1,23, 24]; based on this criterion, all the obtained sequences were assigned to TYLCV and none of them to TYLCSV (Fig. 2).

Pairwise identity analysis allowed us to identify the presence of clusters corresponding to different groups of TYLCD-related genotypes. Considering the complex scenario of the TYLCD-related sequences and the spread of recombinant genomes in the Mediterranean area, including the already known TYLCV-IS76, TYLCV-IS141, TYLCMaV and TYLCAxV recombinants, we decided to more deeply characterize the sequences retrieved in our samples in order to identify possible recombination events and also define the presence or absence of parental strains, such as TYLCV and TYLCV-Mild. Following analyses with the RDP tool, up to four different kinds of recombination events in a single sample could be highlighted. Table 3 presents detailed results for each recombination event, including the associated *P*-values, the start and end points of the events and the putative parental sequences.

**Table 3.** Recombination events identified by RDP in the TYLCD-related sequences Beginning and ending breakpoints are indicated with confidence intervals (CI) at 99%. The probability value indicates the probability of observing a recombination signal, corrected for multiple testing, in that interval. When the minor parent is unknown, the inferred sequence is indicated in brackets. MC indicates multiple comparisons. Methods used to identify the recombination event are R=RDP; G=GENECONV; B=BootScan; M=MaxChi; C=Chimaera; S=SiScan; T=3 Seq; only methods supporting that event are listed. The average *P*-value is the lowest calculated and is referred to the method highlighted in bold.

Sample	Recombination event	Beginning breakpoint (99% CI)	Ending breakpoint (99% CI)	Major parent × minor parent (probability)	Probability value (MC corrected)	Methods	Avg. *P*-value
HPCT_001	1	1998 (1915–2057)	2399 (2296–2490)	TYLCV-Mild (97.2%) × Unknown (TYLCSV)	3.23E−13	RBMCST	6.92E−15
	2	2547 (2486–2745)	2633 (2486–2745)	TYLCV-Mild (97.2%) × Unknown (TYLCSV)	3.39E−06	RMCST	3.00E−05
	3	2767 (2750–19)	60 (48–84)	TYLCV-Mild (97.7%) × TYLCSV (93.5%)	8.48E−07	RGMCT	1.20E−05
	4	2635 (2318–2543)	2700 (2678–2746)	TYLCV (97.8%) × TYLCSV (68.9%)	1.21E−11	RGMCT	1.648−08
HPCT_002	1	2759 (2744–16)	141 (132–153)	TYLCV (99.1%) × TYLCSV (92.3%)	6,967E−21	RGBMCST	6.967E−21
	2	1994 (1911–2047)	2395 (2304–2416)	Mild (97.2%) × Unknown (TYLCSV)	9E−15	RBMCST	3.675E−15
	3	2543 (2499–2551)	2694 (2646–2741)	TYLCV (97.9%) × Unknown (TYLCSV)	8.06E−02	RBMCST	1.759E−11
	4	2761 (2740–20)	60 (48–84)	TYLCV-Mild (97.8%) × TYLCSV (93.6%)	2.30E−06	RGMCT	2.30E−06
D1K8_007	1	2759 (2743–19)	141 (132–154)	TYLCV (99%) × TYLCSV (91.7%)	4.92E−02	RGBMCST	8.57E−18
	2	1999 (1919–2001)	2400 (2348–241)	TYLCV-Mild (97.9%) × Unknown (TYLCSV)	4.30E−17	RGBMCST	9.45E−16
	3	2548 (2487–2556)	2671 (2627–2746)	TYLCV (98%) × unknown (TYLCSV)	5.38E−09	RMCST	3.08E−08
D1K8_009	1	1999 (1917–2053)	2400 (2310–2434)	TYLCV-Mild (97.2%) × Unknown (TYLCSV)	3.73E−15	RBMCST	6.17E−16
	2	2548 (2488–2735)	2634 (2488–2735)	TYLCV (97.1%) × Unknown (TYLCSV)	1.68E−09	RMCST	2.82E−08
	3	2773 (2743–19)	60 (48–84)	TYLCV-Mild (97.6%) × TYLCSV (93.2%)	5.50E−07	RGMCT	1.40E−06
	4	2735 (2480–2544)	2700 (2690–2735)	TYLCV (97.9%) × TYLCSV (70.5%)	1.13E−11	RGMCT	2.67E−06
D1K8_013	1	1994 (1910–2052)	2394	TYLCV-Mild (96.9%) × Unknown (TYLCSV)	8.20E−15	RBMCST	7.85E−15
	2	2548 (2490–2556)	2670 (2628–2735)	TYLCV (97.3%) × Unknown (TYLCSV)	2.37E−13	RGMCST	1.41E−11
	3	2754 (2739–20)	60 (48–84)	TYLCV (98.6%) × TYLCSV (94.9%)	5.54E−07	RGMCT	3.16E−05
D1K8_010	1	2753 (2738–17)	142 (131–152)	TYLCV (99.3%) × TYLCSV (93.2%)	7.28E−05	RGBMCST	8.49E−21

Overall, with the RCA-ONT approach, we detected the known TYLCV-IS141-like recombinant in almost all the tomato fields analysed in the Ragusa province, while the TYLCV-IS76-like recombinant was found only in one field (sample D1K8_013). Interestingly, in all the tomato fields tested, a recombinant molecule putatively having TYLCV-Mild and TYLCSV as major and minor parents, respectively, was also found. In the watermelon field (sample D1K8_010), a TYLCV-IS141-like recombinant was the only TYLCD-related genome identified.

The parental genome sequences of TYLCV and TYLCSV, previously commonly found in Sicily [3,5], could not be detected, supporting the results obtained with SDT (Fig. 2). This could suggest that parental viruses could have been displaced over time by recombinant genomes with increased fitness, at least in the fields considered. Nonetheless, it is worth underlining that the RCA technique can introduce a bias by selectively enriching the dominant molecules present in a sample, making it impossible to exclude a scant occurrence of parental genomes.

### Recombination analysis of samples collected in 1994 and 1999

**Fig. 3.**
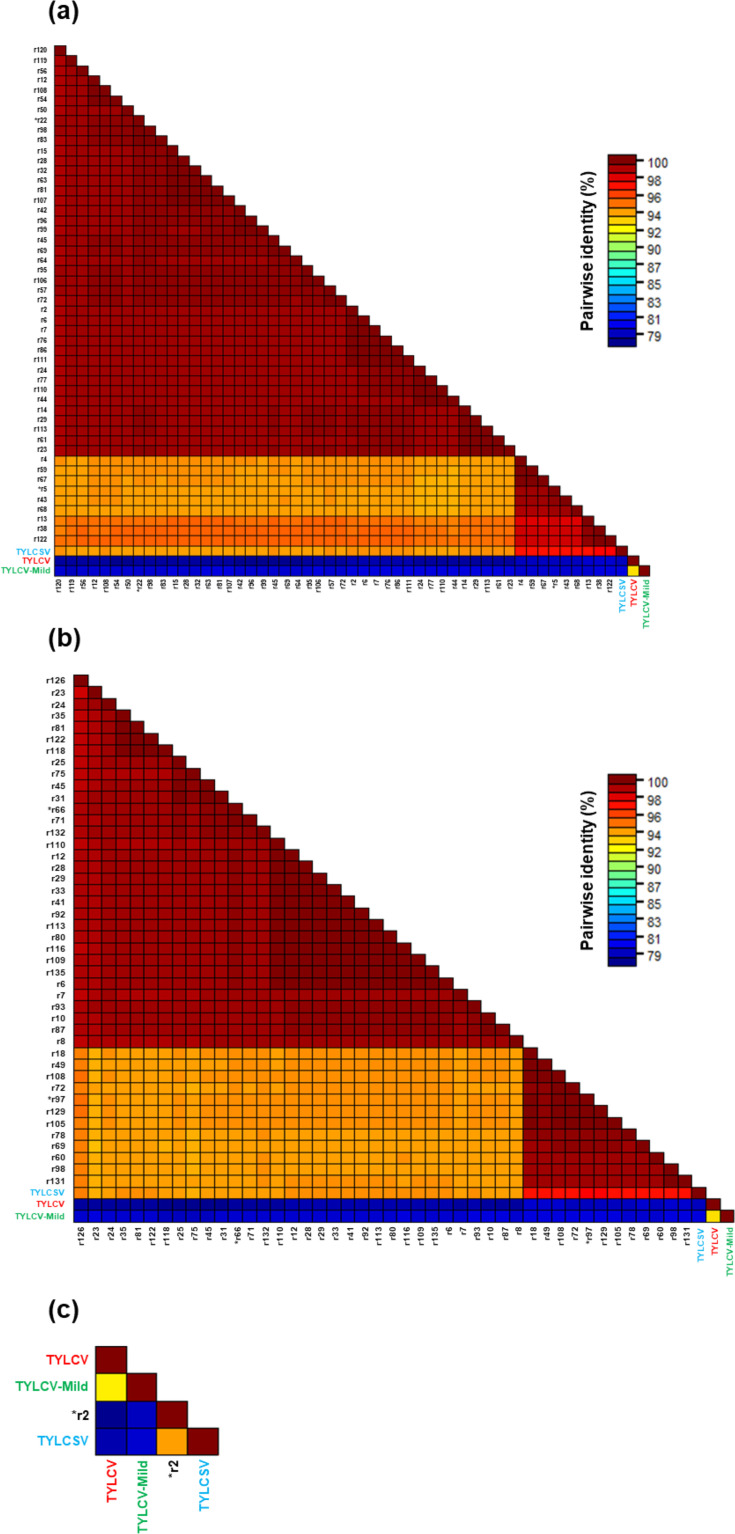
Pairwise identity matrices of TYLCD-related sequences obtained from each sample and the parental sequences from GenBank. Each matrix represents individual samples as follows: (a) D1K8_018, (**b**) D1K8_017 and (c) D1K8_016. Recombinant (**r**) sequences were named with progressive numbers, within each sample. Parental genomes are written with the following colour code: TYLCV in red, TYLCV-Mild in green and TYLCSV in blue. Different clusters are identified at ≥94% identity, the defined value for strain demarcation of begomoviruses. Representative sequences for each cluster used for phylogenetic analysis are indicated with an asterisk.

Thanks to the availability of new powerful sequencing techniques, such as the ONT MinION pipeline, we decided to re-analyse old samples, collected in the same geographical areas, where TYLCSV sequences only were reported [8]. The pairwise identity analysis highlighted the existence of two different clusters of sequences in each of the two tomato samples D1K8_017 and D1K8_018 (Fig. 3). These clusters represent two different groups of TYLCSV-related isolates previously identified in Sicily in 1995 (Z28390) and in 2004 (GU951759). Similarly, the unique sequence obtained from the mixed weed sample showed a high level of identity (99.64%) with the sequence of the Sicilian isolate Z28390.

The results confirmed the sole presence of TYLCSV in that time frame (Fig. 3), supporting the hypothesis that the spread of TYLCV and consequently the onset of TYLCSV/TYLCV recombinants occurred after 1,999, at least in this intensive tomato farming area.

### Characterization of the TYLCV-MILD/TYLCSV recombinant

To investigate the nature of the recombinant molecule identified in the tomato samples from 2022 to 2022, putatively having TYLCV-Mild and TYLCSV as parentals (Table 3), an in-depth characterization was performed. The RDP analysis highlighted the insertion of a fragment of about 54 nt showing 98–100% identity with TYLCSV immediately on the right side of the stem-loop, while the remaining sequence matched with TYLCV-Mild. However, a more detailed analysis conducted with blastn on the genomic portions differentiating TYLCV-Mild from TYLCV indicated that the region between 1,998 and 2,399 nt matched with TYLCV, with an identity of 99%, while the region between 2,415 and 2,755 nt matched with TYLCV-Mild, with an identity of 100%. These results show that the recombinant molecule has a TYLCV backbone with a portion of TYLCV-Mild and one of TYLCSV. Its tripartite nature led us to name it TYLCV-IMS54; the representative sequence derived from sample D1K8_009 (r10 in Fig. 2d) was deposited in GenBank with the accession no. PQ873011.

**Fig. 4.**
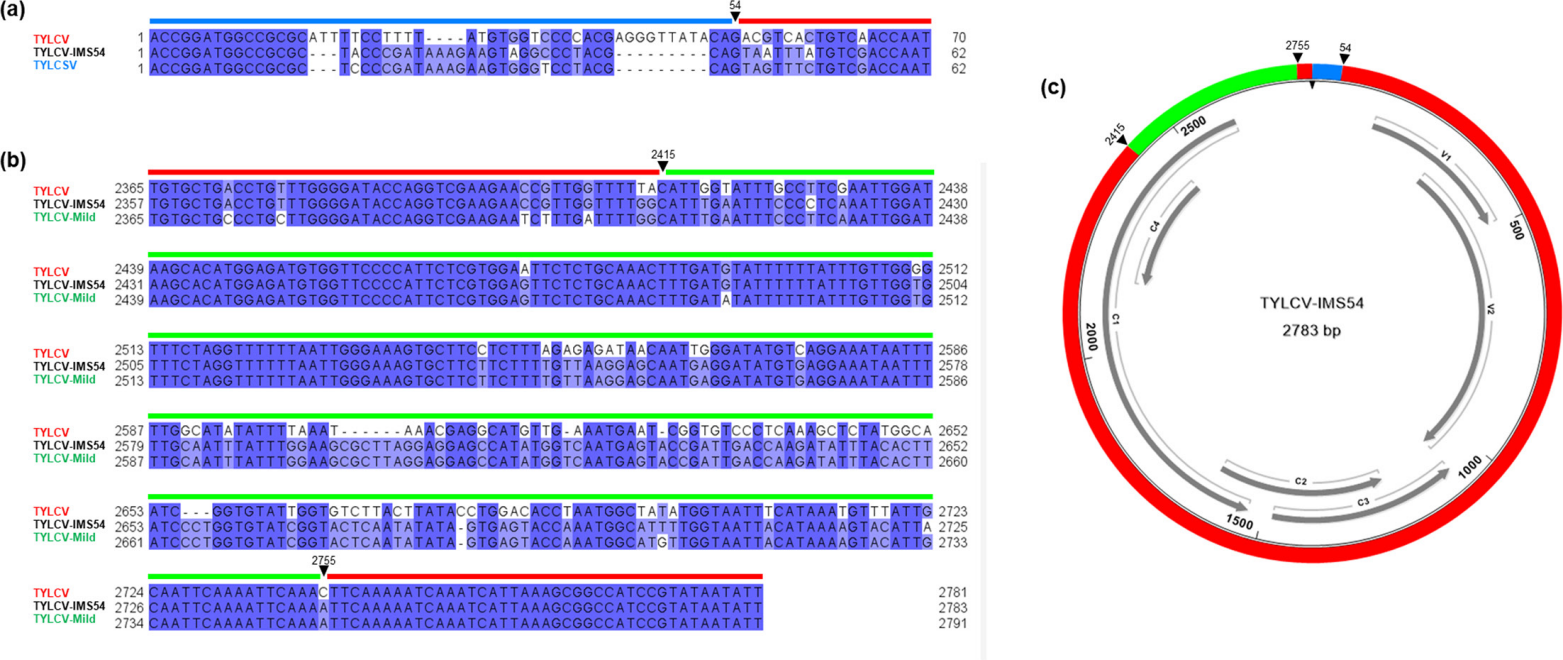
Features of the TYLCV-IMS54 recombinant. (**a**) DNA alignment of sequences of TYLCV (DQ144621, red), TYLCSV (NC_003828, blue) and TYLCV-ISM54 (PQ873011, black) including the first recombination site; (**b**) DNA alignment of sequences of TYLCV (DQ144621, red), TYLCV-Mild (KJ913682, green) and TYLCV-ISM54 (PQ873011, black) including the second and third recombination sites. The black arrow indicates the recombination point. The number above the arrow indicates the coordinate of the recombination site in the recombinant TYLCV-ISM54. (**c**) Schematic representation of the genome of the recombinant TYLCV-ISM54 identified in this study. Parental genomes are indicated with the following colour code: TYLCV in red, TYLCSV in blue and TYLCV-Mild in green.

Recombination events involving TYLCV-Mild and TYLCSV were previously reported on common bean in Almeria (Spain), consisting of the TYLCMaV genome [25]. However, differently from TYLCMaV that included a TYLCSV portion of about 1,130 nt, the recombinant TYLCV-IMS54 is characterized by an extremely reduced TYLCSV contribution (Fig. 4).

PCR analysis with dedicated primers followed by Sanger sequencing confirmed the presence of TYLCV-IMS54 in the original tomato samples (Supplementary File S2).

### Phylogenetic analyses

**Fig. 5.**
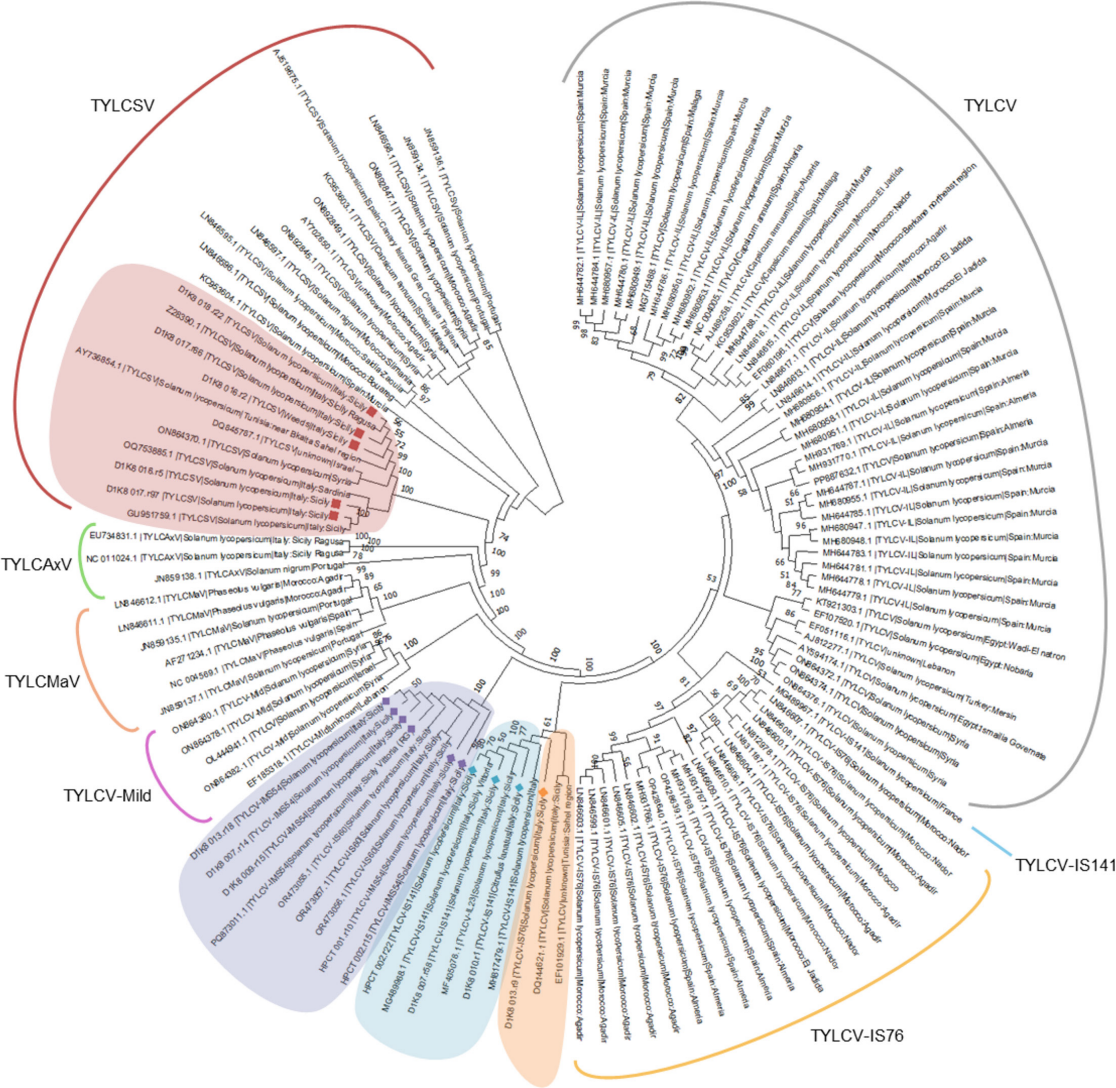
NJ phylogenetic tree of sequences obtained in this work, challenged with full-length TYLCD-related sequences from the Mediterranean area, downloaded from NCBI Virus. For each RCA-ONT sample, a representative sequence was used, named as follows: ‘name of sample’, ‘r’, ‘sequence number’. The sequence PQ873011.1, deposited in GenBank as representative of the TYLCV-IMS54 recombinant, corresponds to the sequence D1K8007.r10. TYLCV/TYLCSV recombinants are indicated by diamonds: TYLCV-IS76, orange; TYLCV-IS141, turquoise; TYLCV-IMS54, purple. TYLCSV parental sequences are indicated by red squares.

Phylogenetic analysis showed that the TYLCV-IS141-like recombinants found in this study cluster together with TYLCV-IS141 recombinant types of Italian origin, but distantly from the unique TYLCV-IS141 isolated in France (MG489967). Similarly, our TYLCV-IS76-like sequence clusters with the TYLCV isolate 8-4/2004 and a TYLCV from Tunisia, rather than with the sequences annotated as TYLCV-IS76 from Morocco and Spain (Fig. 5). These findings likely indicate the existence of independent recombination events in different geographical areas.

The recombinant TYLCV-IMS54 sequences cluster with the TYLCV-IS60 recombinants reported in Sicilian tomato samples in 2024 [11] and recently deposited in the GenBank database (OR473055, OR473056 and OR473057) (Fig. 5). Indeed, our TYLCV-IMS54 type sequence (PQ873011) shows more than 99.7% identity with the three TYLCV-IS60 sequences. In light of this high similarity, a recombination analysis including TYLCV-Mild as a putative parental was performed on the three TYLCV-IS60 sequences mentioned above. The results highlighted that the TYLCV-IMS54/TYLCV-IS60-like sequences represent a new group of recombinants whose genome is composed of the three parentals: TYLCV (major parent), TYLCV-Mild and TYLCSV. Again, the existence of a single clade including all these sequences highlights their independent origin in Sicily (Fig. 5).

## Discussion

MinION sequencing following circular DNA molecules enrichment has emerged as a powerful and affordable tool for characterizing the geminivirus population infecting crops, addressing potential limitations of second-generation sequencing methods [15,16]. Indeed, although the base-calling accuracy of Nanopore technologies still does not match that of second-generation sequencing platforms, their main advantage lies in the ability to achieve greater assembly completeness, owing to longer read lengths that are well suited to the size of geminiviral genomes [15]. In this study, we took advantage of the MinION sequencing technology to investigate the population dynamics of invasive TYLCD-related viruses in Sicily, a region of intensive tomato cultivation.

First of all, we highlighted a shift in the TYLCD*-*associated viral landscape, with the apparent displacement of parental viral genomes over the years in favour of TYLCV recombinants with enhanced fitness, such as TYLCV-IS141- and TYLCV-IS76-like recombinants [26]. Interestingly, the detection of the TYLCV-IMS54/TYLCV-IS60 recombinant in Sicily from as early as 2016 [11] until our 2022 survey suggests that this variant might also exhibit a high level of fitness that deserves further dedicated comparative studies. This recombinant is characterized by the introgression of a portion of 50–60 nt of TYLCSV in the intergenic region, immediately after the stem loop and by a portion of 341 nt originating from TYLCV-Mild on the left side of the genome. The presence of a small portion of TYLCSV could be crucial for increasing fitness, thus reinforcing the conclusions reported by [26]. However, we cannot exclude that the TYLCV-Mild insertion could also have a further role in modulating the pathogenicity, asking for ad hoc investigations.

Among the factors possibly shaping the population dynamics of TYLCD-associated viruses, an important role can be played by the widespread adoption of host genotypes carrying resistance genes, such as *Ty-1*, as previously suggested [26,28]. Advantages in vector transmission provided by viral genome recombinations can also be evoked as evolutionary driving forces, together with concomitant possible evolutionary shift in whitefly populations. In this context, environmental factors such as temperature, rainfall regimes, linked to global climate changes and cultivation practices may also influence the plant-pathogen relationship.

TYLCV-Mild and TYLCSV were reported to infect watermelon and other cucurbits in Jordan, producing leaf yellowing, curling and mottling symptoms [29] and TYLCV was detected in watermelon in Tunisia [30], in squash in Cuba [31], in cucumber in Kuwait [32] and in *Cucurbita maxima* in Japan [33]. To our knowledge, TYLCD-related viruses were never detected in Europe/Italy on cucurbits, and this is the first report of a TYLCV-IS141-like recombinant in Italy. The lack of PCR amplification with *B. tabaci*-specific primers [34] from our sample (data not shown) allowed us to exclude contamination by infected whiteflies. Our findings imply that TYLCV-IS141-like recombinants can infect cucurbit species, further confirming their superior fitness compared to parental genomes.

## Conclusion

In conclusion, our study confirmed the effectiveness of RCA combined with ONT technology as a robust and cost-effective approach for characterizing geminivirus populations in the field, enabling real-time monitoring of their spread. Through this technology, we documented a dynamic shift in the TYLCD-associated viral population in Sicily, dominated by TYLCV recombinants such as TYLCV-IS141-, TYLCV-IS76- and TYLCV-IMS54/IS60-like variants. Our findings underline the adaptive advantage of recombination and emphasize the importance of continuous monitoring in order to develop effective management strategies and mitigate future risks for cultivation.

## Supplementary material

10.1099/mgen.0.001529 Fig. S1.

10.1099/mgen.0.001529 Fig. S2.

## Abbreviations

LAMP: loop-mediated isothermal amplification
NJ: neighbour-joining
RCA: rolling circle amplification
RDP: Recombination Detection Program
SDT: sequence demarcation tool
TYLCD: tomato yellow leaf curl disease
TYLCSV: tomato yellow leaf curl Sardinia virus
TYLCV: tomato yellow leaf curl virus
