# Supplementary material for: Oxford nanopore sequencing as a useful tool for investigating the population dynamics of invasive begomoviruses in Sicily
Source: Microb Genom. 2025 Nov 3;11(11):001529. doi: 10.1099/mgen.0.001529 (PMC12582396; doi:10.1099/mgen.0.001529)
Supplement: Fig. S2. [file mgen-11-01529-s002.pdf]

# Supplementary File S2

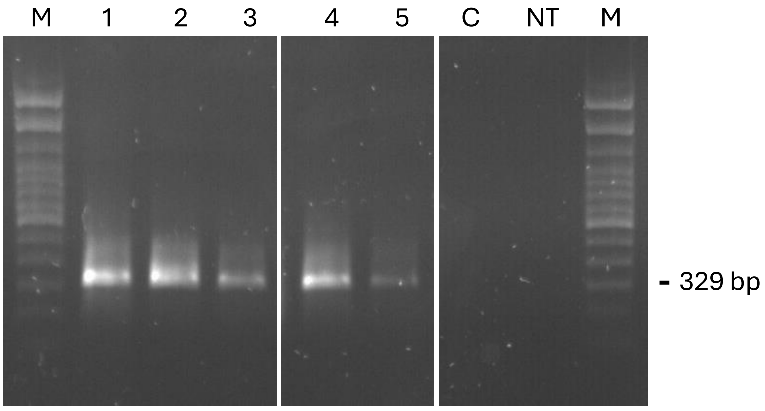


Supplementary File S2 - Validation by PCR of the presence of TYLCV-IMS54 recombinant in tomato samples collected in 2020-2022 and used for RCA-ONT analysis. The length of the TYLCV-IMS54 recombinant amplicon at 329 bp is highlighted. Lanes are as follows: 1) HPCT001, 2) HPCT002, 3) D1K8013, 4) D1K8007, 5) D1K8009, C) Heathy tomato plant, NT) No template control, M) 100 bp ladder.
